# Fractional exhaled nitric oxide distribution and its relevant factors in the general adult population and its healthy subpopulation

**DOI:** 10.1016/j.jacig.2024.100253

**Published:** 2024-04-08

**Authors:** Mitsuhiro Yamada, Masato Takase, Kumi Nakaya, Tomohiro Nakamura, Mana Kogure, Naoki Nakaya, Naoya Fujino, Tsutomu Tamada, Chikashi Iwasaki, Manami Suzuki, Shuichiro Matsumoto, Nobuo Fuse, Akira Uruno, Kazuki Kumada, Soichi Ogishima, Shinichi Kuriyama, Masakazu Ichinose, Hisatoshi Sugiura, Atsushi Hozawa

**Affiliations:** a Department of Respiratory Medicine, Tohoku University Graduate School of Medicine, Sendai, Japan; b Department of Preventive Medicine and Epidemiology, Tohoku Medical Megabank Organization, Tohoku University, Sendai, Japan; c Kyoto Women’s University, Kyoto, Japan; d Department of Integrative Genomics, Tohoku Medical Megabank Organization, Tohoku University, Sendai, Japan; e Department of Biobank, Tohoku Medical Megabank Organization, Tohoku University, Sendai, Japan; f Department of Health Record Informatics, Tohoku Medical Megabank Organization, Tohoku University, Sendai, Japan; g Osaki Citizen Hospital Academic Center, Osaki, Japan

**Keywords:** Fractional exhaled nitric oxide, asthma, COPD, biomarkers, health survey of residents

## Abstract

**Background:**

Measurement of fractional exhaled nitric oxide (Feno) has been used in the diagnosis and management of asthma. Understanding the distribution of Feno in a larger resident population and its “healthy” subpopulation would contribute to the interpretation of Feno in clinical practice.

**Objective:**

This study aimed to investigate the distribution and its associated factors in the adult population and its healthy subpopulations.

**Methods:**

We conducted a cross-sectional study of 8,638 men and 17,288 women aged 20 years or older living in Miyagi prefecture, Japan. We investigated the distribution of Feno and its associated factors in all subjects, a subpopulation with no history of upper and lower airway diseases (healthy subpopulation 1), and a subpopulation with no history of upper and lower airway diseases, normal lung function, and no positivity for other biomarkers of type 2 inflammation (healthy subpopulation 2).

**Results:**

The distribution of Feno in healthy subpopulations, especially in healthy subpopulation 2 (median [interquartile range], 17 [12-23] with 95th percentile of 36 ppb) was lower than in all subjects (19 [13-26] ppb with 95th percentile of 47 ppb). In healthy subpopulation 1, 10.3% had elevated Feno (≥35 ppb), and elevated Feno was positively associated with factors including obstructive ventilatory defect, blood eosinophilia, house dust mite–specific IgE positivity, and history of hypertension. Male sex was associated with elevated Feno in all subjects and healthy subpopulations.

**Conclusion:**

The distribution of Feno in the healthy subpopulation supports the validity of the criteria (≥35 ppb) currently used in Japan for the diagnosis of asthma.

Fractional exhaled nitric oxide (Feno) is a useful indicator for estimating the presence of type 2 airway inflammation because type 2 inflammation and its related cytokines, IL-4 and IL-13, increase nitric oxide production in the lungs, especially airway epithelium.^1^, ^2^, ^3^, ^4^, ^5^ Therefore, the measurement of Feno is a helpful tool that can assess type 2 inflammation in the airways to aid in the diagnosis of asthma, monitor type 2 airway inflammation in patients undergoing asthma treatment, and detect asthma overlapping with chronic obstructive pulmonary disease (COPD).^6^, ^7^, ^8^, ^9^ The 2011 American Thoracic Society (ATS) guidelines set Feno cutoff values of 25 and 50 ppb, with interpretations according to eosinophilic airway inflammation and responsiveness to steroid therapy.^10^ In Japan, the cutoff value of Feno (≥35 ppb) was suggested by previous Japanese studies as appropriate for the adjunctive diagnosis of asthma.^11^, ^12^, ^13^ Previous reports also suggested that various factors such as smoking status, sinus disease, and allergic rhinitis may influence Feno.^11^^,^^12^^,^^14^^,^^15^

We sought to elucidate the distribution of Feno in a large adult resident population and its “healthy” subpopulation (that is, with no history of upper and lower airway diseases that could influence Feno, such as asthma, allergic rhinitis, or chronic sinusitis) so we could contribute to the interpretation of Feno in clinical practice. We were also interested in how many people had elevated Feno and the characteristics surrounding it, including lung function parameters, in an adult healthy subpopulation. Therefore, in the present study, we examined Feno in a population of about 25,000 Japanese adults in the Tohoku Medical Megabank Community–based Cohort Study (TMM CommCohort Study).^16^^,^^17^ We then investigated the distribution of Feno and its associated factors in a general adult population. Further, to figure out the distribution of Feno in healthy adult population, we examined the distribution in 2 subpopulations: one with no history of upper and lower airway diseases, including asthma, allergic rhinitis, and chronic sinusitis, termed healthy subpopulation 1; and the other with no history of upper or lower airway disease, normal lung function, and no positivity for other biomarkers of type 2 inflammation, termed healthy subpopulation 2. We also examined associated factors, including respiratory function parameters, smoking status, medical history, and other biomarkers of type 2 inflammation, to elucidate the characteristics of subjects with elevated Feno levels in healthy subpopulations.

## Methods

### Study design and subjects

This cross-sectional study was conducted using data from the TMM CommCohort Study; details of the TMM CommCohort Study have been published previously.^16^, ^17^, ^18^ The study included men and women aged 20 years and older living in Miyagi prefecture, northeastern Japan. The study was approved by the institutional ethics committee of the Tohoku Medical Megabank Organization (approval 2023-4-076).

Detailed methods are provided in the Methods section in this article’s Online Repository at www.jaci-global.org. Briefly, data from 29,383 people who underwent Feno measurement and spirometry at a follow-up examination were used in this study. We excluded data from those who withdrew from the study by January 17, 2022; those who did not return the self-report questionnaire; those who did not undergo lung function testing; and those with missing data for height, weight, smoking history, white blood cell count, peripheral blood eosinophil count, specific IgE (sIgE) to house dust mite (HDM; *Dermatophagoides farinae*), and sIgE to cedar pollen (n = 3,457). Therefore, only data from 25,926 participants were analyzed.

### Assessment of Feno

Feno was measured using a NIOX VERO device (Circassia, Uppsala, Sweden). Measurements were performed according to the measurement guidelines jointly proposed by the ATS and the European Respiratory Society (ERS)^19^ and the official statement of the Japanese Respiratory Society for Feno measurement and interpretation.^13^

We examined the distribution of Feno in (1) all participants, (2) healthy subpopulation 1 (participants without a history of airway diseases, including asthma, COPD, chronic bronchitis, other respiratory diseases, allergic rhinitis, and chronic sinusitis), and (3) healthy subpopulation 2 (participants without a history of airway diseases, restrictive or obstructive ventilatory defects, or positivity for type 2 biomarkers, including sIgE to HDM and cedar pollen and elevated blood eosinophil count [≥150 μL^−1^]).

Feno was categorized into the following 4 groups according to ATS/ERS^10^ and Japanese Respiratory Society^13^ guidelines: <25 ppb, 25-34 ppb, 35-49 ppb, and ≥50 ppb. According to the cutoff value for the ancillary diagnosis of asthma, suggested by previous articles in Japan,^11^^,^^13^ Feno ≥35 ppb was considered elevated.

### Assessment of lung function

To calculate the percentage predicted FEV_1_ or vital capacity (%FEV_1_ or %VC), which are values of the lung function measures adjusted for age, sex, and height, we used the reference spirometry values for Japanese adults calculated using the LMS (lambda, mu, and sigma) method.^20^ A restrictive ventilatory defect was defined as %VC of less than 80%. An obstructive ventilatory defect was defined as ratio of FEV_1_ to forced VC (FVC) of less than 0.7.

### Statistical analysis

Data are presented as means (standard deviations [SDs]) or medians (interquartile ranges [IQRs]) for continuous variables, and as numbers (%) for categorical variables. For the characteristics of the 4 subgroups classified according to Feno level, a trend test was performed for continuous variables using a simple linear model to assess the linear association. We also performed a chi-square test to compare the characteristics of categorical variables between Feno subgroups. To analyze the association between elevated Feno (≥35 ppb) and potentially associated factors, multivariate logistic analysis was performed to calculate odds ratios (ORs) and 95% confidence intervals (CIs). Receiver operating characteristic (ROC) analysis was performed to investigate the cutoff value of Feno that discriminates self-reported asthmatic subjects from healthy subjects (healthy subpopulation 2). The cutoff value was determined by the point on the ROC curve closest to the top left. *P* < .05 was considered significant. All analyses were performed by R 4.1.2 software (R Project; www.r-project.org).

## Results

### Characteristics of participants

Table I shows the characteristics of the study participants. A total of 8,638 men and 17,288 women met the inclusion criteria. The mean (SD) age of the study participants was 63.3 (11.7) years. Overall, 4.3% of participants had a lung function test with %VC less than 80 and 4.0% had an FEV_1_/FVC of less than 70%; 65.6% were never smokers, 25.8% were ex-smokers, and 8.6% were current smokers. The median [IQR] peripheral blood eosinophil count was 110 [66-180] μL^−1^. The sIgE positivity to HDM was 17.9% and to cedar pollen was 40.5%. According to the self-report questionnaire regarding current medical history, 2,759 participants (5.3%) were diagnosed with asthma, 4,049 (15.6%) with allergic rhinitis, and 1,101 (4.2%) with chronic sinusitis.

**Table I:** Characteristics of participants according to asthma and smoking status

Characteristic	Never smoker		Ex-smoker		Current smoker		All participants
	Asthma	No asthma	Asthma	No asthma	Asthma	No asthma	
No. of subjects	863	16,145	395	6292	128	2103	25,926
Male sex	103 (11.9)	2410 (14.9)	225 (57.0)	4533 (72.0)	48 (37.5)	1319 (62.7)	8638 (33.3)
Age (years)	60.2 (12.6)	63.7 (11.4)	60.1 (12.7)	64.5 (11.3)	51.0 (12.0)	58.3 (12.6)	63.3 (11.7)
Height (cm)	156.5 (7.0)	156.1 (7.1)	162.3 (7.6)	163.7 (7.3)	161.0 (7.4)	163.7 (7.8)	158.7 (8.1)
Weight (kg)	57.7 (10.6)	56.4 (9.9)	64.0 (11.6)	63.9 (10.5)	62.6 (12.2)	63.0 (12.1)	58.9 (10.9)
BMI (kg/m^2^)	23.5 (3.8)	23.1 (3.5)	24.3 (3.9)	23.8 (3.2)	24.1 (4.2)	23.4 (3.7)	23.3 (3.5)
Underweight	42 (4.9)	951 (5.9)	9 (2.3)	195 (3.1)	4 (3.1)	127 (6.0)	1328 (5.1)
Normal weight	558 (64.7)	11165 (69.2)	243 (61.5)	4138 (65.8)	80 (62.5)	1391 (66.1)	17575 (67.8)
Overweight/obesity	263 (30.5)	4029 (25.0)	143 (36.2)	1959 (31.1)	44 (34.4)	585 (27.8)	7023 (27.1)
%VC (%)	100.4 (13.5)	103.2 (13.7)	101.0 (12.4)	102.0 (13.5)	98.6 (13.3)	100.7 (12.9)	102.6 (13.6)
%VC < 80	54 (6.3)	641 (4.0)	14 (3.5)	298 (4.7)	12 (9.4)	93 (4.4)	1112 (4.3)
FEV_1_ (L)	2.2 (0.6)	2.2 (0.5)	2.4 (0.6)	2.6 (0.6)	2.5 (0.7)	2.7 (0.6)	2.4 (0.6)
%FEV_1_ (%)	98.9 (15.9)	105.8 (15.1)	92.0 (17.1)	97.8 (15.2)	91.4 (15.9)	95.3 (15.2)	102.5 (15.8)
FVC (L)	2.7 (0.7)	2.8 (0.6)	3.2 (0.7)	3.3 (0.7)	3.2 (0.8)	3.4 (0.7)	3.0 (0.7)
%FVC (%)	99.7 (8.3)	102.9 (6.5)	97.0 (10.3)	101.4 (7.6)	95.9 (9.5)	99.1 (8.3)	102.0 (7.2)
FEV_1_/FVC (%)	79.5 (6.9)	81.3 (5.3)	77.0 (8.7)	79.4 (6.2)	78.0 (8.6)	78.9 (7.4)	80.5 (6.0)
FEV_1_/FVC < 0.7	68 (7.9)	303 (1.9)	72 (18.2)	382 (6.1)	16 (12.5)	198 (9.4)	1039 (4.0)
Feno (ppb)	22 [15, 38]	18 [13, 25]	25 [17, 41]	21 [15, 30]	18 [11, 30]	15 [11, 23]	19 [13, 26]
<25 ppb	482 (55.9)	11861 (73.5)	196 (49.6)	3875 (61.6)	89 (69.5)	1680 (79.9)	18183 (70.1)
25-34 ppb	133 (15.4)	2698 (16.7)	60 (15.2)	1362 (21.6)	11 (8.6)	251 (11.9)	4515 (17.4)
35-49 ppb	104 (12.1)	1062 (6.6)	58 (14.7)	712 (11.3)	13 (10.2)	117 (5.6)	2066 (8.0)
≥50.0 ppb	144 (16.7)	524 (3.2)	81 (20.5)	343 (5.5)	15 (11.7)	55 (2.6)	1162 (4.5)
White blood cell count (μL^−1^)	5400 [4600, 6350]	5300 [4500, 6200]	5700 [4850, 6700]	5500 [4600, 6400]	6200 [5200, 7625]	6200 [5300, 7300]	5400 [4600, 6400]
Eosinophils (%)	2.7 [1.5, 4.3]	1.9 [1.1, 3.0]	3.0 [1.8, 4.9]	2.2 [1.3, 3.5]	2.4 [1.4, 4.3]	2.2 [1.3, 3.6]	2.0 [1.2, 3.3]
Eosinophil count (μL^−1^)	139 [80, 237]	100 [60, 161]	168 [98, 283]	120 [72, 196]	160 [90, 283]	141 [82, 223]	110 [66, 180]
HDM sIgE positive	329 (38.1)	2526 (15.6)	167 (42.3)	1091 (17.3)	68 (53.1)	450 (21.4)	4631 (17.9)
Cedar pollen sIgE positive	451 (52.3)	6294 (39.0)	214 (54.2)	2605 (41.4)	72 (56.2)	874 (41.6)	10510 (40.5)
History of disease							
COPD	4 (0.5)	14 (0.1)	10 (2.5)	43 (0.7)	1 (0.8)	3 (0.1)	75 (0.3)
Chronic bronchitis	31 (3.6)	99 (0.6)	14 (3.5)	33 (0.5)	2 (1.6)	9 (0.4)	188 (0.7)
Other respiratory disease	8 (0.9)	126 (0.8)	7 (1.8)	1.2 (73)	2 (1.6)	9 (0.4)	225 (0.9)
Allergic rhinitis	344 (39.9)	2290 (14.2)	158 (40.0)	955 (15.2)	48 (37.5)	254 (12.1)	4049 (15.6)
Chronic sinusitis	97 (11.2)	618 (3.8)	46 (11.6)	262 (4.2)	14 (10.9)	64 (3.0)	1101 (4.2)
Atopic dermatitis	111 (12.9)	514 (3.2)	46 (11.6)	166 (0.6)	28 (21.9)	81 (3.9)	946 (3.6)
Hypertension	219 (25.4)	4018 (24.9)	120 (30.4)	2056 (32.7)	19 (14.8)	486 (23.1)	6918 (26.7)
Diabetes	34 (3.9)	719 (4.5)	28 (7.1)	508 (8.1)	10 (7.8)	128 (6.1)	1427 (5.5)
Dyslipidemia	169 (19.6)	2439 (15.1)	64 (16.2)	838 (13.3)	11 (8.6)	162 (7.7)	3683 (14.2)
Hyperuricemia	13 (1.5)	191 (1.2)	20 (5.1)	392 (6.2)	1 (0.8)	81 (3.9)	698 (2.7)
Stroke	20 (2.3)	289 (1.8)	7 (1.8)	208 (3.3)	3 (2.3)	43 (2.0)	570 (2.2)
Myocardial infarction	27 (3.1)	365 (2.3)	18 (4.6)	353 (5.6)	3 (2.3)	48 (2.3)	814 (3.1)

Values are expressed as mean (SD) or median [IQR] for continuous variables or as nos. (%) for categorical variables. Underweight was defined as BMI <18.5 kg/m^2^; normal weight, BMI 18.5-24.9 kg/m^2^; and overweight/obesity, BMI ≥25.0 kg/m^2^.

### Feno distribution in general population and healthy subpopulation

**Fig 1:**
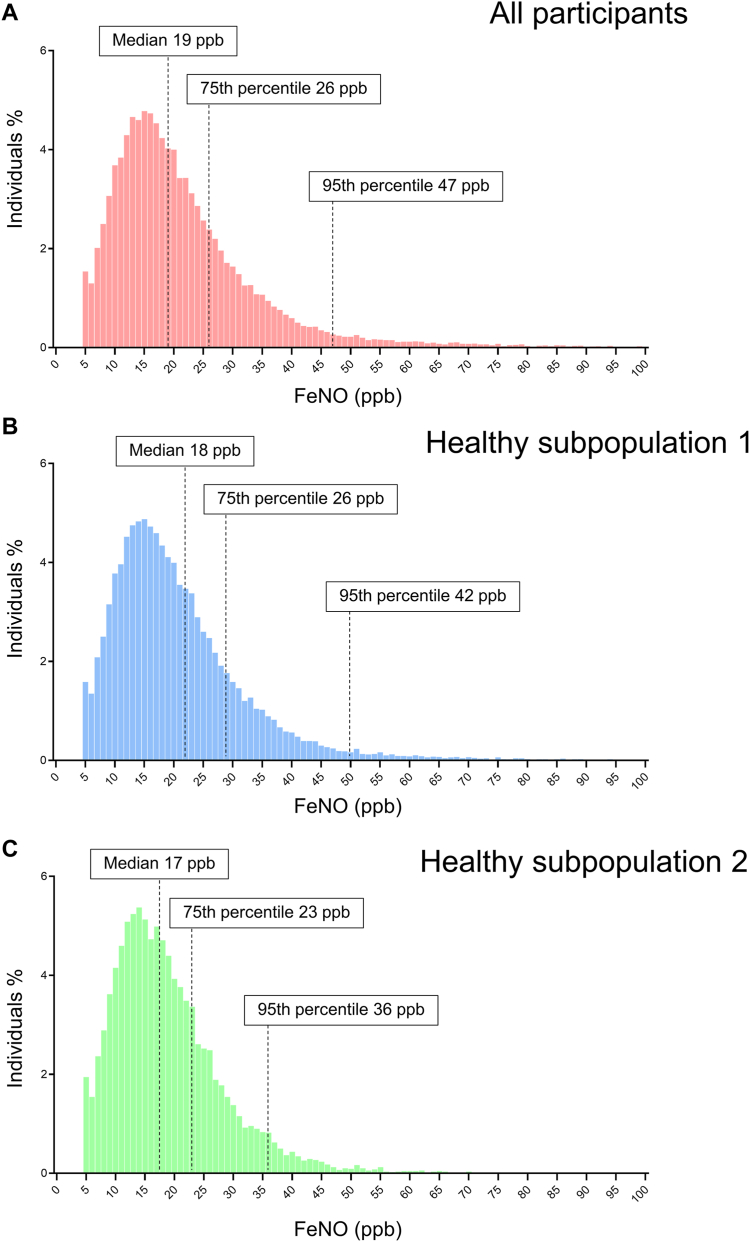
Distribution of Feno in **(A)** all participants studied (n = 25,926), **(B)** healthy subpopulation 1 (n = 20,137), and **(C)** healthy subpopulation 2 (n = 8,321).

The median [IQR] value of Feno for all participants studied was 19 [13-26] ppb, with a 95th percentile of 47 ppb (Fig 1, *A*). Feno was elevated in the participants with a history of asthma compared to those without a diagnosis of asthma, regardless of smoking status, whereas Feno decreased in current smokers compared to never smokers or ex-smokers, regardless of asthma diagnosis (Table I), as previously reported.^11^^,^^12^^,^^15^

We stratified the subjects into 4 subgroups according to Feno level and compared characteristics, including physical and laboratory characteristics, smoking history, and diagnostic history, between subgroups (Table II). A total of 70.1% of the participants had Feno levels <25 ppb; 17.4% had 25.0-34.9 ppb; 8.0% had 35.0-49.9 ppb; and 4.5% had ≥50 ppb. The subgroup with elevated Feno levels had more male subjects; lower %VC, %FEV_1_, and FEV_1_/FVC; a higher percentage of subjects with FEV_1_/FVC of less than 70%; fewer never smokers and current smokers; and more ex-smokers. Laboratory values in the subgroup with elevated Feno showed higher eosinophil counts and higher sIgE positivity to HDM and cedar pollen. Regarding medical history, asthma, allergic rhinitis, and chronic sinusitis were highly prevalent.

**Table II:** Comparisons of subgroup characteristics according to Feno level

Characteristic	All participants	Feno				*P* value
		<25 ppb	25-34 ppb	35-49 ppb	≥50 ppb	
No. of subjects	25926	18183	4515	2066	1162	
Male sex	8638 (33.3)	5040 (27.7)	2000 (44.3)	1056 (51.1)	542 (46.6)	<.001
Age (years)	63.3 (11.7)	62.6 (11.9)	65.4 (1.6)	65.0 (11.2)	62.2 (12.7)	<.001
Height (cm)	158.7 (8.1)	157.9 (7.8)	16.0 (8.3)	161.1 (8.4)	161.1 (8.3)	<.001
Weight (kg)	58.9 (10.9)	57.9 (10.7)	6.9 (1.9)	62.2 (10.7)	61.6 (11.1)	<.001
BMI (kg/m^2^)	23.3 (3.5)	23.2 (3.5)	23.7 (3.4)	23.9 (3.3)	23.7 (3.5)	<.001
Underweight	1328 (5.1)	1077 (5.9)	139 (3.1)	76 (3.7)	36 (3.1)	<.001
Normal weight	17575 (67.8)	12491 (68.7)	3008 (66.6)	1305 (63.2)	771 (66.4)	
Overweight/obesity	7023 (27.1)	4615 (25.4)	1368 (3.3)	685 (33.2)	355 (30.6)	
Smoking status						<.001
Never smoker	17008 (65.6)	12343 (67.9)	2831 (62.7)	1166 (56.4)	668 (57.5)	
Ex-smoker	6687 (25.8)	4071 (22.4)	1422 (31.5)	770 (37.3)	424 (36.5)	
Current smoker						
1-19 cigarettes per day	1462 (5.6)	1133 (6.2)	178 (3.9)	93 (4.5)	58 (5.0)	
≥20 cigarettes per day	769 (3.0)	636 (3.5)	84 (1.9)	37 (1.8)	12 (1.0)	
%VC (%)	102.6 (13.6)	102.7 (13.6)	102.6 (13.6)	102.2 (13.6)	101.3 (13.1)	.001
%VC < 80	1112 (4.3)	763 (4.2)	195 (4.3)	97 (4.7)	57 (4.9)	.513
FEV_1_ (L)	2.4 (0.6)	2.4 (0.6)	2.4 (0.6)	2.5 (0.6)	2.4 (0.6)	<.001
%FEV_1_ (%)	102.5 (15.8)	103.5 (15.6)	101.4 (15.6)	99.5 (16.2)	96.5 (16.8)	<.001
FVC (L)	3.0 (0.7)	2.9 (0.7)	3.0 (0.7)	3.1 (0.7)	3.1 (0.8)	<.001
%FVC (%)	102.0 (7.2)	102.2 (7.0)	102.1 (7.1)	101.5 (8.1)	99.2 (8.7)	<.001
FEV_1_/FVC (%)	80.5 (6.0)	80.9 (5.8)	8.0 (5.7)	79.6 (6.5)	78.3 (7.0)	<.001
FEV_1_/FVC < 0.7	1039 (4.0)	612 (3.4)	180 (4.0)	125 (6.1)	122 (10.5)	<.001
Feno (ppb)	19 [13, 26]	15 [11, 19]	28 [26, 31]	39 [36, 43]	65 [55, 83]	
White blood cell count (μL^−1^)	5400 [4600, 6400]	5400 [4500, 6300]	5400 [4600, 6400]	5500 [4700, 6500]	5600 [4800, 6575]	<.001
Eosinophils (%)	2.0 [1.2, 3.3]	1.9 [1.1, 3.0]	2.2 [1.3, 3.5]	2.7 [1.7, 4.1]	4.0 [2.5, 6.3]	<.001
Eosinophil count (μL^−1^)	109 [66, 180]	100 [60, 161]	120 [71, 191]	143 [89, 230]	225 [137, 350]	<.001
HDM sIgE positive	4631 (17.9)	2601 (14.3)	910 (2.2)	585 (28.3)	535 (46.0)	<.001
Cedar pollen sIgE positive	10510 (40.5)	6947 (38.2)	1876 (41.6)	990 (47.9)	697 (60.0)	<.001
History of disease						
Asthma	1386 (5.3)	767 (4.2)	204 (4.5)	175 (8.5)	240 (20.7)	<.001
COPD	75 (0.3)	39 (0.2)	13 (0.3)	10 (0.5)	13 (1.1)	<.001
Chronic bronchitis	188 (0.7)	120 (0.7)	31 (0.7)	16 (0.8)	21 (1.8)	<.001
Other respiratory disease	225 (0.9)	152 (0.8)	39 (0.9)	20 (1.0)	14 (1.2)	.574
Allergic rhinitis	4049 (15.6)	2512 (13.8)	720 (15.9)	419 (20.3)	398 (34.3)	<.001
Chronic sinusitis	1101 (4.2)	709 (3.9)	177 (3.9)	97 (4.7)	118 (10.2)	<.001
Atopic dermatitis	946 (3.6)	598 (3.3)	159 (3.5)	84 (4.1)	105 (9.0)	<.001
Hypertension	6917 (26.7)	4572 (25.1)	1332 (29.5)	681 (33.0)	332 (28.6)	<.001
Diabetes	1427 (5.5)	937 (5.2)	297 (6.6)	137 (6.6)	56 (4.8)	<.001
Dyslipidemia	3683 (14.2)	2598 (14.3)	647 (14.3)	291 (14.1)	147 (12.7)	.477
Hyperuricemia	698 (2.7)	395 (2.2)	160 (3.5)	89 (4.3)	54 (4.6)	<.001
Stroke	570 (2.2)	374 (2.1)	124 (2.7)	49 (2.4)	23 (2.0)	.036
Myocardial infarction	814 (3.1)	506 (2.8)	179 (4.0)	77 (3.7)	52 (4.5)	<.001

Values are expressed as mean (SD) or median [IQR] for continuous variables or as nos. (%) for categorical variables. Underweight was defined as BMI <18.5 kg/m^2^; normal weight, BMI 18.5-24.9 kg/m^2^; and overweight/obesity, BMI ≥25.0 kg/m^2^.

We then performed multivariate logistic analysis to search for factors independently associated with elevated Feno (≥35 ppb) in all participants studied (Table III). The results showed that factors associated with elevated Feno were male sex (OR [95% CI] 1.48 [1.29-1.68]), being tall (170-179 cm, 1.22 [1.03-1.45]; 180-190 cm, 1.44 [1.16-1.80]; ≥190 cm, 2.18 [1.36-3.40]), 50 kg range (1.22 [1.07-1.39]), 60 kg range (1.45 [1.26-1.67]), 70 kg range (1.48 [1.25-1.76]), 80 kg range (1.32 [1.03-1.69]), FEV_1_/FVC less than 0.7 (1.54 [1.30-1.82]), peripheral blood eosinophilia (150-299 μL^−1^, 1.95 [1.79-2.13]; ≥300 μL^−1^, 3.95 [3.53-4.43]), positive for HDM sIgE (2.38 [2.16-2.62]), cedar pollen sIgE (1.23 [1.13-1.34]), history of asthma (2.20 [1.92-2.53]), allergic rhinitis [1.44 [1.30-1.60]), chronic sinusitis (1.29 [1.08-1.52]), and hypertension (1.13 [1.04-1.24]) were all significantly associated with elevated Feno levels. In contrast, being a current smoker (1-19 cigarettes per day, 0.57 [0.46-0.68]; ≥20 cigarettes per day, 0.27 [0.20-0.37]) was significantly negatively associated with having elevated Feno. The association between underweight and overweight was analyzed separately from that between height and weight, which could not be analyzed simultaneously because body mass index (BMI) is calculated from height and weight and cannot be independent of them. Nevertheless, being overweight was significant (1.15 [1.05-1.25]), and it correlated with elevated Feno levels (Table IV).

**Table III:** Logistic multivariable regression analysis for factors (including height and weight) associated with elevated Feno levels (≥35 ppb) by population

Factor	OR (95% CI) for:		
	All subjects	Healthy subpopulation 1	Healthy subpopulation 2
Age category			
20-29 years	1.00 (ref)	1.00 (ref)	1.00 (ref)
30-39 years	0.73 (0.46-1.18)	0.66 (0.35-1.29)	0.35 (0.05-2.82)
40-49 years	0.69 (0.45-1.11)	0.68 (0.38-1.31)	0.36 (0.08-2.57)
50-59 years	0.86 (0.56-1.38)	0.91 (0.51-1.71)	0.68 (0.18-4.47)
60-69 years	1.15 (0.75-1.83)	1.19 (0.68-2.23)	1.27 (0.35-8.22)
70-79 years	1.33 (0.86-2.11)	1.37 (0.78-2.58)	1.82 (0.50-11.80)
80+ years	1.30 (0.77-2.23)	1.20 (0.62-2.44)	1.89 (0.47-12.91)
Male sex	1.47 (1.29-1.68)	1.66 (1.41-1.95)	2.05 (1.50-2.81)
Height			
<150 cm	1.00 (ref)	1.00 (ref)	1.00 (ref)
150-169 cm	1.05 (0.91-1.22)	0.98 (0.83-1.18)	0.83 (0.62-1.13)
170-179 cm	1.22 (1.03-1.46)	1.13 (0.91-1.39)	0.97 (0.66-1.42)
180-189 cm	1.44 (1.16-1.80)	1.32 (1.02-1.71)	1.35 (0.84-2.17)
190+ cm	2.18 (1.37-3.41)	1.84 (1.04-3.13)	1.96 (0.70-4.96)
Weight			
<50 kg	1.00 (ref)	1.00 (ref)	1.00 (ref)
50-59 kg	1.22 (1.07-1.39)	1.25 (1.06-1.48)	1.28 (0.94-1.75)
60-69 kg	1.45 (1.26-1.67)	1.50 (1.25-1.80)	1.59 (1.14-2.25)
70-79 kg	1.48 (1.25-1.76)	1.51 (1.22-1.87)	1.57 (1.04-2.37)
80-89 kg	1.32 (1.03-1.69)	1.46 (1.08-1.95)	2.32 (1.33-3.98)
90+ kg	1.41 (0.96-2.04)	1.57 (0.97-2.46)	2.70 (0.93-6.79)
Smoking status			
Never smoker	1.00 (ref)	1.00 (ref)	1.00 (ref)
Ex-smoker	1.04 (0.94-1.15)	1.03 (0.91-1.16)	1.09 (0.86-1.38)
Current smoker			
1-19 cigarettes per day	0.57 (0.46-0.68)	0.51 (0.40-0.64)	0.43 (0.22-0.78)
≥20 cigarettes per day	0.27 (0.20-0.37)	0.30 (0.21-0.42)	0.56 (0.24-1.12)
Lung function impairment			
%VC < 80	0.99 (0.82-1.19)	0.93 (0.74-1.17)	—
FEV_1_/FVC < 0.7	1.54 (1.30-1.82)	1.56 (1.27-1.92)	—
Blood eosinophil count			
<150 μL^−1^	1.00 (ref)	1.00 (ref)	
150-299 μL^−1^	1.95 (1.79-2.13)	1.79 (1.61-1.99)	—
≥300 μL^−1^	3.95 (3.53-4.43)	3.06 (2.64-3.54)	—
HDM sIgE positive	2.38 (2.16-2.62)	2.40 (2.12-2.70)	—
Cedar pollen sIgE positive	1.23 (1.13-1.34)	1.31 (1.18-1.45)	—
History of disease			
Asthma	2.20 (1.92-2.53)	—	—
COPD	1.29 (0.74-2.19)	—	—
Allergic rhinitis	1.44 (1.30-1.60)	—	—
Chronic sinusitis	1.29 (1.08-1.52)	—	—
Atopic dermatitis	1.18 (0.97-1.42)	1.29 (0.95-1.73)	1.23 (0.47-2.68)
Hypertension	1.13 (1.04-1.24)	1.13 (1.01-1.25)	0.99 (0.81-1.20)
Diabetes	0.88 (0.74-1.03)	0.93 (0.76-1.12)	1.22 (0.87-1.68)
Dyslipidemia	0.86 (0.76-0.96)	0.85 (0.74-0.99)	0.91 (0.69-1.17)
Hyperuricemia	1.05 (0.85-1.29)	1.01 (0.78-1.29)	1.17 (0.69-1.89)
Stroke	0.78 (0.59-1.01)	0.85 (0.62-1.14)	1.07 (0.62-1.73)
Myocardial infarction	0.98 (0.79-1.20)	1.04 (0.82-1.32)	1.15 (0.72-1.75)

**Table IV:** Logistic multivariable regression analysis for factors (including BMI) associated with elevated Feno levels (≥35 ppb) by population

Factor	OR (95% CI) for:		
	All subjects	Healthy subpopulation 1	Healthy subpopulation 2
Age category			
20-29 years	1.00 (ref)	1.00 (ref)	1.00 (ref)
30-39 years	0.73 (0.46-1.18)	0.65 (0.35-1.28)	0.28 (0.04-2.18)
40-49 years	0.69 (0.44-1.10)	0.67 (0.38-1.29)	0.29 (0.07-2.02)
50-59 years	0.85 (0.55-1.35)	0.88 (0.50-1.66)	0.54 (0.15-3.47)
60-69 years	1.08 (0.70-1.70)	1.10 (0.63-2.06)	0.92 (0.27-5.76)
70-79 years	1.18 (0.77-1.86)	1.22 (0.70-2.28)	1.22 (0.36-7.68)
80+ years	1.09 (0.65-1.86)	1.01 (0.53-2.05)	1.18 (0.31-7.76)
Male sex	1.97 (1.78-2.18)	2.17 (1.93-2.45)	2.89 (2.29-3.65)
BMI			
Normal weight	1.00 (ref)	1.00 (ref)	1.00 (ref)
Underweight	0.89 (0.72-1.09)	1.22 (0.94-1.60)	1.17 (0.70-2.13)
Overweight	1.15 (1.05-1.25)	1.42 (1.08-1.88)	1.45 (0.85-2.68)
Smoking status			
Never smoker	1.00 (ref)	1.00 (ref)	1.00 (ref)
Ex-smoker	1.06 (0.96-1.17)	1.04 (0.92-1.18)	1.09 (0.86-1.38)
Current smoker			
1-19 cigarettes per day	0.57 (0.47-0.69)	0.51 (0.40-0.65)	0.43 (0.22-0.78)
≥20 cigarettes per day	0.28 (0.20-0.37)	0.30 (0.21-0.42)	0.57 (0.25-1.14)
Lung function impairment			
%VC < 80	0.97 (0.80-1.17)	0.92 (0.73-1.15)	—
FEV_1_/FVC < 0.7	1.54 (1.30-1.82)	1.56 (1.26-1.92)	—
Blood eosinophil count			
<150 μL^−1^	1.00 (ref)	1.00 (ref)	
150-299 μL^−1^	1.97 (1.80-2.15)	1.80 (1.62-2.00)	—
≥300 μL^−1^	3.94 (3.52-4.42)	3.06 (2.64-3.54)	—
HDM sIgE positive	2.37 (2.15-2.61)	2.39 (2.12-2.70)	—
Cedar pollen sIgE positive	1.24 (1.14-1.35)	1.31 (1.19-1.45)	—
History of disease			
Asthma	2.19 (1.90-2.51)	—	—
COPD	1.30 (0.75-2.21)	—	—
Allergic rhinitis	1.44 (1.29-1.59)	—	—
Chronic sinusitis	1.29 (1.08-1.52)	—	—
Atopic dermatitis	1.18 (0.97-1.42)	1.28 (0.94-1.72)	1.18 (0.45-2.56)
Hypertension	1.14 (1.04-1.25)	1.13 (1.01-1.26)	1.01 (0.83-1.22)
Diabetes	0.87 (0.74-1.03)	0.93 (0.76-1.12)	1.21 (0.87-1.67)
Dyslipidemia	0.86 (0.76-0.96)	0.86 (0.74-0.99)	0.93 (0.71-1.20)
Hyperuricemia	1.05 (0.85-1.29)	1.00 (0.78-1.28)	1.18 (0.70-1.90)
Stroke	0.79 (0.60-1.03)	0.87 (0.63-1.16)	1.06 (0.62-1.72)
Myocardial infarction	0.97 (0.78-1.18)	1.03 (0.81-1.31)	1.12 (0.71-1.71)

Underweight was defined as BMI <18.5 kg/m^2^; normal weight, BMI 18.5-24.9 kg/m^2^; and overweight/obesity, BMI ≥25.0 kg/m^2^.

To determine the distribution of Feno in our healthy subjects, we then examined the distribution of Feno in healthy subpopulation 1 (n = 20,137). Their median [IQR] Feno level was 18 [13-26] ppb, with a 95th percentile of 42 ppb (Fig 1, *B,* and Table V). A total of 72.2% of healthy subpopulation 1 participants had Feno levels <25 ppb, 17.4% had 25.0-34.9 ppb, 7.3% had 35.0-49.9 ppb, and 3.0% had ≥50 ppb. In healthy subpopulation 1, the subgroup with elevated Feno levels had more male subjects, lower %FEV_1_ and FEV_1_/FVC, a higher percentage of subjects with FEV_1_/FVC less than 70%, fewer never smokers and current smokers, more ex-smokers, higher eosinophil counts, and higher sIgE positivity to HDM and cedar pollen (Table V). Multivariate logistic analysis showed that factors associated with elevated Feno were similar in all participants studied, including male sex (OR [95% CI] 1.66 [1.41-1.95]), obesity (1.42 [1.08-1.88]), FEV_1_/FVC less than 0.7 (1.56 [1.27-1.92]), peripheral blood eosinophilia (150-299 μL^−1^, 1.79 [1.61-1.99]; ≥300 μL^−1^, 3.06 [2.64-3.54]), positive for HDM sIgE (2.40 [2.12-2.70]), cedar pollen sIgE (1.31 [1.18-1.45]), history of hypertension (1.13 [1.01-1.25]), and current smoking (1-19 cigarettes per day, 0.51 [0.40-0.64]; ≥20 cigarettes per day, 0.30 [0.21-0.42]) (Tables III and IV).

**Table V:** Comparisons of characteristics between subgroups classified by Feno in healthy subpopulation 1

Characteristic	All participants	Feno				*P* value
		<25 ppb	25-34 ppb	35-49 ppb	≥50 ppb	
No. of subjects	20,137	14,543 (72.2)	3510 (17.4)	1478 (7.3)	606 (3.0)	
Male sex	6982 (34.7)	4210 (28.9)	1633 (46.5)	827 (56.0)	312 (51.5)	<.001
Age (years)	64.2 (11.2)	63.4 (11.5)	66.4 (9.9)	66.6 (9.9)	64.6 (11.5)	<.001
Height (cm)	158.6 (8.1)	157.9 (7.9)	160.0 (8.3)	161.4 (8.4)	161.1 (8.6)	<.001
Weight (kg)	59.0 (10.8)	58.0 (10.6)	60.9 (10.8)	62.7 (10.5)	61.8 (11.5)	<.001
BMI (kg/m^2^)	23.4 (3.5)	23.2 (3.5)	23.7 (3.3)	24.0 (3.3)	23.7 (3.6)	<.001
Underweight	1001 (5.0)	832 (5.7)	105 (3.0)	46 (3.1)	18 (3.0)	<.001
Normal weight	13629 (67.7)	9975 (68.6)	2326 (66.3)	932 (63.1)	396 (65.3)	
Overweight/obesity	5597 (27.3)	3736 (25.7)	1079 (30.7)	500 (33.8)	192 (31.7)	
Smoking status						<.001
Never smoker	13279 (65.9)	9908 (68.1)	2202 (62.7)	820 (55.5)	349 (57.6)	
Ex-smoker	5065 (25.2)	3189 (21.9)	1094 (31.2)	559 (37.8)	223 (36.8)	
Current smoker						
1-19 cigarettes per day	1147 (5.7)	912 (6.3)	142 (4.0)	67 (4.5)	26 (4.3)	
≥20 cigarettes per day	646 (3.2)	534 (3.7)	72 (2.1)	32 (2.2)	8 (1.3)	
%VC (%)	102.7 (13.7)	102.7 (13.7)	102.5 (13.6)	102.4 (13.5)	101.9 (13.6)	.082
%VC <80	872 (4.3)	619 (4.3)	155 (4.4)	67 (4.5)	31 (5.1)	.728
FEV_1_ (L)	2.4 (0.6)	2.3 (0.6)	2.4 (0.6)	2.5 (0.6)	2.4 (0.6)	<.001
%FEV_1_ (%)	102.8 (15.8)	103.6 (15.7)	101.6 (15.6)	99.8 (16.1)	98.6 (16.9)	<.001
FVC (L)	2.9 (0.7)	2.9 (0.7)	3.0 (0.7)	3.1 (0.8)	3.1 (0.8)	<.001
%FVC (%)	102.3 (7.0)	102.3 (6.9)	102.3 (6.9)	101.9 (8.0)	100.7 (8.3)	<.001
FEV_1_/FVC (%)	80.5 (5.8)	80.8 (5.7)	80.0 (5.6)	79.5 (6.3)	79.0 (6.6)	<.001
FEV_1_/FVC < 0.7	736 (3.7)	469 (3.2)	133 (3.8)	88 (6.0)	46 (7.6)	<.001
Feno (ppb)	18 [13, 26]	15 [11, 19]	28 [26, 31]	39 [36, 43]	61 [54, 75]	
White blood cell count (μL^−1^)	5400 [4600, 6400]	5400 [4500, 6300]	5400 [4600, 6400]	5500 [4700, 6500]	5500 [4700, 6400]	<.001
Eosinophils (%)	2.0 [1.2, 3.1]	1.8 [1.1, 2.9]	2.2 [1.3, 3.4]	2.5 [1.5, 4.0]	3.5 [2.1, 5.2]	<.001
Eosinophil count (μL^−1^)	102 [62, 172]	99 [60, 159]	118 [70, 189]	133 [81, 219]	189 [114, 291]	<.001
HDM sIgE positive	2760 (13.7)	1655 (11.4)	553 (15.8)	315 (21.3)	237 (39.1)	<.001
Cedar pollen sIgE positive	6814 (33.8)	4681 (32.2)	1227 (35.0)	580 (39.2)	326 (53.8)	<.001
History of disease						
Atopic dermatitis	457 (2.3)	334 (2.3)	65 (1.9)	32 (2.2)	26 (4.3)	.003
Hypertension	5518 (27.4)	3771 (25.9)	1053 (30.0)	499 (33.8)	195 (32.2)	<.001
Diabetes	1147 (5.7)	773 (5.3)	233 (6.6)	103 (7.0)	38 (6.3)	.002
Dyslipidemia	2678 (13.3)	1954 (13.4)	476 (13.6)	183 (12.4)	65 (10.7)	.171
Hyperuricemia	515 (2.6)	316 (2.2)	110 (3.1)	62 (4.2)	27 (4.5)	<.001
Stroke	456 (2.3)	308 (2.1)	94 (2.7)	37 (2.5)	17 (2.8)	.151
Myocardial infarction	625 (3.1)	395 (2.7)	139 (4.0)	58 (3.9)	33 (5.4)	<.001

Values are expressed as mean (SD) or median [IQR] for continuous variables or as nos. (%) for categorical variables. Underweight was defined as BMI <18.5 kg/m^2^; normal weight, BMI 18.5-24.9 kg/m^2^; and overweight/obesity, BMI ≥25.0 kg/m^2^.

We further investigated the distribution of the subpopulation without a history of airway disease, a restrictive ventilatory defect (%VC < 80), an obstructive ventilatory defect (FEV_1_/FVC less than 0.7), and positivity of type 2 biomarkers (sIgE to the HDM and cedar pollen, and elevated blood eosinophil count [≥150 μL^−1^])—that is, healthy subpopulation 2—because we thought that the participants, who had no history of airway diseases but had a defect found on lung function testing and/or positivity of other type 2 biomarkers might have an undiagnosed airway disease that could influence Feno levels. We excluded these participants from the study population and examined the distribution of healthy subpopulation 2 (n = 8,321; Table VI). The median [IQR] value of Feno for healthy subpopulation 2 was 17 [12-23] ppb, with a 95th percentile of 36 ppb (Fig 1, *C,* and Table VI). A total of 78.1% of healthy subpopulation 1 had Feno levels <25 ppb, 15.5% had 25.0-34.9 ppb, 5.3% had 35.0-49.9 ppb, and 1.2% had ≥50 ppb. Multivariate logistic analysis showed that factors associated with elevated Feno in healthy subpopulation 2 were male sex (OR [95% CI] 2.05 [1.50-1.81]), 60 kg range (1.59 [1.14-2.25]), 70 kg range (1.57 [1.04-2.37]), 80 kg range (2.32 [1.33-3.98]), and current smoker (1-19 cigarettes per day, 0.43 [0.22-0.78]) (Tables III and IV).

**Table VI:** Comparison of characteristics between subgroups classified by Feno in healthy subpopulation 2

Characteristic	All participants	Feno				*P* value
		<25 ppb	25-34 ppb	35-49 ppb	≥50 ppb	
No. of subjects	8321	6496 (78.1)	1286 (15.5)	439 (5.3)	100 (1.2)	
Male sex	2388 (28.7)	1545 (23.8)	544 (42.3)	242 (55.1)	57 (57.0)	<.001
Age (years)	65.8 (10.2)	65.0 (10.6)	68.1 (8.3)	69.0 (7.8)	70.8 (6.1)	<.001
Height (cm)	157.4 (7.9)	156.9 (7.6)	158.8 (8.2)	160.7 (8.8)	159.9 (9.1)	<.001
Weight (kg)	57.7 (10.3)	56.9 (10.1)	59.8 (10.5)	62.0 (10.4)	61.7 (12.3)	<.001
BMI (kg/m^2^)	23.2 (3.4)	23.1 (3.4)	237 (3.3)	24.0 (3.2)	24.0 (3.8)	<.001
Underweight	397 (4.8)	348 (5.4)	35 (2.79	11 (2.5)	3 (3.0)	<.001
Normal weight	5789 (69.6)	4572 (70.4)	869 (67.6)	290 (66.1)	58 (58.0)	
Overweight/obesity	2135 (25.7)	1576 (24.3)	382 (29.7)	138 (31.4)	39 (39.0)	
Smoking status						<.001
Never smoker	6014 (72.3)	4811 (74.1)	885 (68.8)	261 (59.5)	57 (57.0)	
Ex-smoker	1803 (21.7)	1247 (19.2)	354 (27.5)	162 (36.9)	40 (40.0)	
Current smoker						
1-19 cigarettes per day	350 (4.2)	307 (4.7)	32 (2.5)	10 (2.3)	1 (1.0)	
≥20 cigarettes per day	154 (1.9)	131 (2.0)	15 (1.2)	6 (1.4)	2 (2.0)	
%VC (%)	104.7 (12.4)	104.7 (12.4)	104.7 (12.5)	104.6 (12.1)	105.4 (12.1)	.918
FEV_1_ (L)	2.3 (0.5)	2.3 (0.5)	2.4 (0.5)	2.5 (0.6)	2.5 (0.6)	<.001
%FEV_1_ (%)	106.4 (14.5)	106.8 (14.5)	105.2 (14.2)	104.2 (14.2)	106.7 (16.8)	<.001
FVC (L)	2.9 (0.6)	2.9 (0.6)	3.0 (0.7)	3.1 (0.7)	3.1 (0.7)	<.001
%FVC (%)	103.2 (6.0)	103.1 (6.0)	103.2 (6.0)	103.6 (6.2)	104.4 (7.1)	.028
FEV_1_/FVC (%)	81.0 (4.9)	81.2 (4.9)	80.4 (4.8)	80.4 (4.9)	80.7 (5.2)	<.001
Feno (ppb)	17 [12, 23]	15 [11, 19]	28 [26, 30]	38 [36, 42]	55 [52, 64]	
White blood cell count (μL^−1^)	5200 [4400, 6100]	5200 [4400, 6100]	5300 [4500, 6200]	5300 [4500, 6200]	5300 [4575, 6225]	.214
Eosinophils (%)	1.4 [0.9, 2.0]	1.4 [0.9, 2.0]	1.4 [1.0, 2.0]	1.6 [1.1, 2.1]	1.7 [1.0, 2.3]	<.001
Eosinophil count (μL^−1^)	73 [49, 102]	72 [48, 102]	78 [50, 108]	82 [58, 112]	96 [47, 121]	<.001
History of disease						
Atopic dermatitis	121 (1.5)	104 (1.6)	11 (0.9)	4 (0.9)	2 (2.0)	.151
Hypertension	2347 (28.2)	1760 (27.1)	397 (30.9)	146 (33.3)	44 (44.0)	<.001
Diabetes	463 (5.6)	323 (5.0)	93 (7.2)	37 (8.4)	10 (10.0)	<.001
Dyslipidemia	1222 (14.7)	939 (14.5)	207 (16.1)	59 (13.4)	17 (17.0)	.349
Hyperuricemia	149 (1.8)	94 (1.4)	35 (2.79	15 (3.4)	5 (5.0)	<.001
Stroke	193 (2.3)	136 (2.1)	39 (3.0)	12 (2.7)	6 (6.0)	.014
Myocardial infarction	237 (2.8)	163 (2.5)	49 (3.8)	16 (3.6)	9 (9.0)	<.001

Values are expressed as mean (SD) or median [IQR] for continuous variables or as nos. (%) for categorical variables. Underweight was defined as BMI <18.5 kg/m^2^; normal weight, BMI 18.5-24.9 kg/m^2^; and overweight/obesity, BMI ≥25.0 kg/m^2^.

Because both sex and smoking status were independently associated with elevated Feno in all subjects and in healthy subpopulations, we also examined the distribution in subgroups divided by sex and smoking status (Table VII). In healthy subpopulation 2, the Feno levels were higher in men than in women, irrespective of smoking status.

**Table VII:** Distribution of Feno classified by sex and smoking status by population

Population	Sex	Smoking status		
		Never smoker	Ex-smoker	Current smoker
All subjects	Male	23 [17-32] (10-56)	23 [16-31] (10-49)	22 [16-29.0] (10-40)
	Female	23 [17-32] (10-57)	23 [16-31] (10-49)	21 [15-28] (9-43)
Healthy subpopulation 1	Male	17 [12-24] (7-44)	16 [11-24] (7-42)	16 [10-21] (6-35)
	Female	17 [12-24] (7-43)	17 [12-23] (7-39)	16 [12-22] (7-33)
Healthy subpopulation 2	Male	17 [12-24] (7-48)	16 [12-22] (7-40)	15 [10-20] (6-33)
	Female	14 [10-20] (6-39)	13 [9-19] (6-33)	12 [8-16] (6-27)

Values are expressed as median [IQR] (5th-95th percentile).

To investigate the cutoff value of Feno that discriminates those with self-reported asthma from healthy subjects, we performed ROC analysis using data from the 8321 individuals who were assumed to be healthy (healthy subpopulation 2; Table VI) and the 1386 individuals who self-reported asthma (Table I). ROC analysis of data from all individuals revealed that the cutoff value of Feno that discriminated between these 2 groups was 58 ppb (area under the receiver operating characteristic curve = 0.650, sensitivity = 0.56, specificity = 0.65; see Fig E1 and Table E1 in the Online Repository at www.jaci-global.org). We also performed ROC analyses in the subgroups classified by sex and smoking history. The cutoff value in each subgroup is as follows: male never smokers, 68 ppb; male ex-smokers, 67 ppb; male current smokers, 61 ppb; female never smokers, 53 ppb; female ex-smokers, 37 ppb; and female current smokers, 31 ppb (Fig E1 and Table E1).

## Discussion

In this study, we investigated Feno in a large Japanese adult population and analyzed the distribution of Feno levels and factors associated with Feno levels. In addition, to estimate the distribution of Feno levels in a healthy adult population, we analyzed the subpopulation with no history of upper or lower respiratory disease (healthy subpopulation 1), and the subpopulation with no history of upper or lower respiratory disease, normal lung function, and no positivity of other type 2 biomarkers (healthy subpopulation 2). The results showed that Feno levels were lower in healthy subpopulations 1 and 2 than in all subjects. In particular, the distribution of Feno in healthy subpopulation 2 was almost identical to that reported in a previous and relatively small healthy Japanese population.^12^ Our study suggests that the currently used indicator, Feno ≥35 ppb for elevated Feno in Japan according to previous studies,^11^^,^^12^ is reasonable for a diagnostic aid for asthma and asthma-COPD overlap.

Of all the subjects in this study, 12.5% had elevated Feno (≥35 ppb), although only 12.9% of this population with elevated Feno had been diagnosed with asthma by a physician. To characterize the population with elevated Feno in a healthy adult population, we then performed an analysis in healthy subpopulations. The results showed that 10.3% of healthy subpopulation 1 had elevated Feno (≥35 ppb). In this population, male sex, BMI, obstructive ventilatory defect, peripheral blood eosinophilia, HDM sIgE positivity, cedar pollen sIgE positivity, and history of hypertension were significantly positively associated with elevated Feno, whereas current smoking was negatively associated. The population with elevated Feno in healthy subpopulation 1 is a symptom-free population with no history of upper or lower respiratory tract disease, albeit self-reported. However, there were more subjects with an obstructive ventilatory defect than those in the low Feno population, as well as more subjects with peripheral blood eosinophilia, as well as more HDM sIgE–positive subjects and cedar pollen sIgE–positive subjects. These facts may suggest that the subjects with elevated Feno in healthy subpopulation 1 may be partially confounded by those with undiagnosed upper and lower airway diseases that can cause elevated Feno, including asthma. To clarify the characteristics of these elevated subjects, we plan to follow them for development and changes in lung function over time.

In the meantime, to estimate the distribution of Feno and its related factors in a healthy population under more stringent conditions for the reasons mentioned above, we performed analysis in a healthy subpopulation 2 and found that 6.5% had elevated Feno levels (≥35 ppb). In this population, male sex and some weight ranges were significantly positively associated with elevated Feno levels, and current smoking was negatively associated with elevated Feno levels. On the one hand, there are consistent reports of a negative association with current smoking, and a mechanism has been suggested.^21^^,^^22^ On the other hand, there are conflicting reports regarding sex, with some reporting an association^15^ and others reporting no association.^12^^,^^14^ In the present analysis of a relatively large population, male sex was independently and positively associated with elevated Feno levels, even after adjustment for smoking status and body size, which differed between men and women. The results of this study regarding sex differences are not consistent with the biological studies showing estrogen action on inducible nitric oxide synthase activity^23^, ^24^, ^25^ and that an increase in androgen receptors is associated with a decrease in Feno.^26^ Although the differences in their distribution indices, such as median, were small and unlikely to have a significant impact on actual clinical practice decisions, further investigation is needed to determine whether male sex is mechanistically associated with higher Feno.

Obesity was positively associated with elevated Feno in our analysis of a population including healthy individuals, in contrast to results in the asthmatic population,^27^, ^28^, ^29^ especially in late-onset asthma.^27^ This association between obesity and elevated Feno was no longer significant in healthy subpopulation 2, but the results suggest an associated trend (Table IV). Similar to our study, previous reports in nonasthmatic children^30^ and adults^31^ have reported a positive association between obesity and Feno. This may suggest that in healthy subjects without type 2 inflammation, unlike in people with asthma, obesity might increase Feno without mediating the mechanism of type 2 inflammation. However, further biochemical analysis is needed to elucidate the exact mechanism.

Healthy subpopulation 2 had 6.5% with high Feno levels (≥35 ppb), indicating that Feno levels are still distributed in the high range despite this population’s being assumed to be healthy and without type 2 inflammation. This observation suggests that Feno levels are influenced not only by the presence of type 2 inflammation but also by other factors,^32^ including the microbiome,^33^, ^34^, ^35^ airway S-nitrosothiol concentration,^36^ airway pH,^37^, ^38^, ^39^ and genetic background.^18^^,^^40^ These facts, including our findings, again remind us that Feno is only an adjunctive diagnostic tool in the diagnosis of asthma.

In the present study, we found a weak but independent association between elevated Feno and hypertension in all subjects and in healthy subpopulation 1, although there was no association in healthy subpopulation 2. Like a previous study that reported an association between allergic rhinitis and hypertension risk,^41^ our results may suggest an unexplored pathomechanism between type 2 inflammation and hypertension.

We performed an ROC analysis of data from individuals who were considered healthy (healthy subpopulation 2) and individuals who self-reported asthma. The cutoff value of Feno was high, with lower area under the receiver operating characteristic curve, sensitivity, and specificity compared to a previous report.^11^ This is probably because those self-reporting asthma in our study were likely to include patients receiving treatment, including inhaled corticosteroid therapy, and both the median and range of Feno levels were lower (Table I) than those previously reported in untreated asthma.^11^

This study has several strengths. First, it is the largest ever cohort of Feno and spirometry in a voluntary population of community residents, including healthy subjects. Second, it is a valuable data set in a generally understudied ethnic population. However, there are several limitations. First, the information on participants’ medical history is based on self-report through questionnaires, which may not reflect reality. However, to account for this possibility, this study attempted to select healthy participants using spirometry, eosinophil count, and sIgE in addition to self-reported medical history. The distribution of Feno results was almost identical to that previously reported in a small but healthy Japanese population screened by medical examination.^12^ Second, the study subjects were adults aged 20 years or older living in Miyagi prefecture, and only those who agreed to participate in the study were included, so their demographic composition differed from the actual resident population,^42^ with more women and a higher mean age. The values for physical indicators, laboratory values, and disease prevalence may also differ from those of the actual resident population. To account for these biases, we performed a multivariable analysis to search for factors associated with high Feno levels after adjusting for explanatory factors. However, we cannot exclude the possibility of bias that cannot be fully adjusted for in the multivariate analysis. In addition, the data on the distribution of Feno may differ from the actual resident population.

In conclusion, we analyzed the distribution of Feno levels in a larger Japanese adult population than previously reported. We also analyzed their distribution in the subpopulation without upper and lower airway disease (healthy subpopulation 1) and in the subpopulation with no upper and lower airway disease, normal lung function, and negative biomarkers of type 2 inflammation (healthy subpopulation 2) to estimate the distribution of Feno levels in healthy adults. The distribution of Feno levels in healthy subpopulation 2, using more stringent criteria, was consistent with previous results in the healthy subpopulation, supporting the validity of the criteria for high Feno levels currently used in Japan. While high Feno levels were correlated with factors such as asthma, eosinophilia, and sIgE positivity, consistent with previous reports, male sex was independently and positively correlated with elevated Feno levels in all subjects and in the healthy subpopulations.

## Disclosure statement

Supported by grants from the 10.13039/501100001691Japan Society for the Promotion of Science (JSPS; Grant-in-Aid for Science Research, 19K10637 to T.N., 23K07594 to M.Y., and 20H03684 to S.H.); Tohoku Medical Megabank Project from the Ministry of Education, Culture, Sports, Science, and Technology (MEXT); the 10.13039/100009619Japan Agency for Medical Research and Development (AMED; JP21tm0124005); and JST SPRING (grant JPMJSP2114 to M.T.). This research used the supercomputer system provided by the Tohoku Medical Megabank Project founded by AMED (grant JP21tm0424601).

Disclosure of potential conflict of interest: The authors declare that they have no relevant conflicts of interest.

**Clinical implications**

Feno levels in the healthy subpopulation of Japanese adult resident population suggest that the currently indicator of high Feno (≥35 ppb) used in Japan to aid in diagnosis of asthma is reasonable.

## Supplementary data

Supplementary Fig E1 A

Supplementary Fig E1 B

Supplementary Fig E1 C

Supplementary Fig E1 D

Supplementary Fig E1 E

Supplementary Fig E1 F

Supplementary Fig E1 G
