# Supplementary figures and images for: Fractional exhaled nitric oxide distribution and its relevant factors in the general adult population and its healthy subpopulation
Source: J Allergy Clin Immunol Glob. 2024 Apr 8;3(3):100253. doi: 10.1016/j.jacig.2024.100253 (PMC11090912; doi:10.1016/j.jacig.2024.100253)

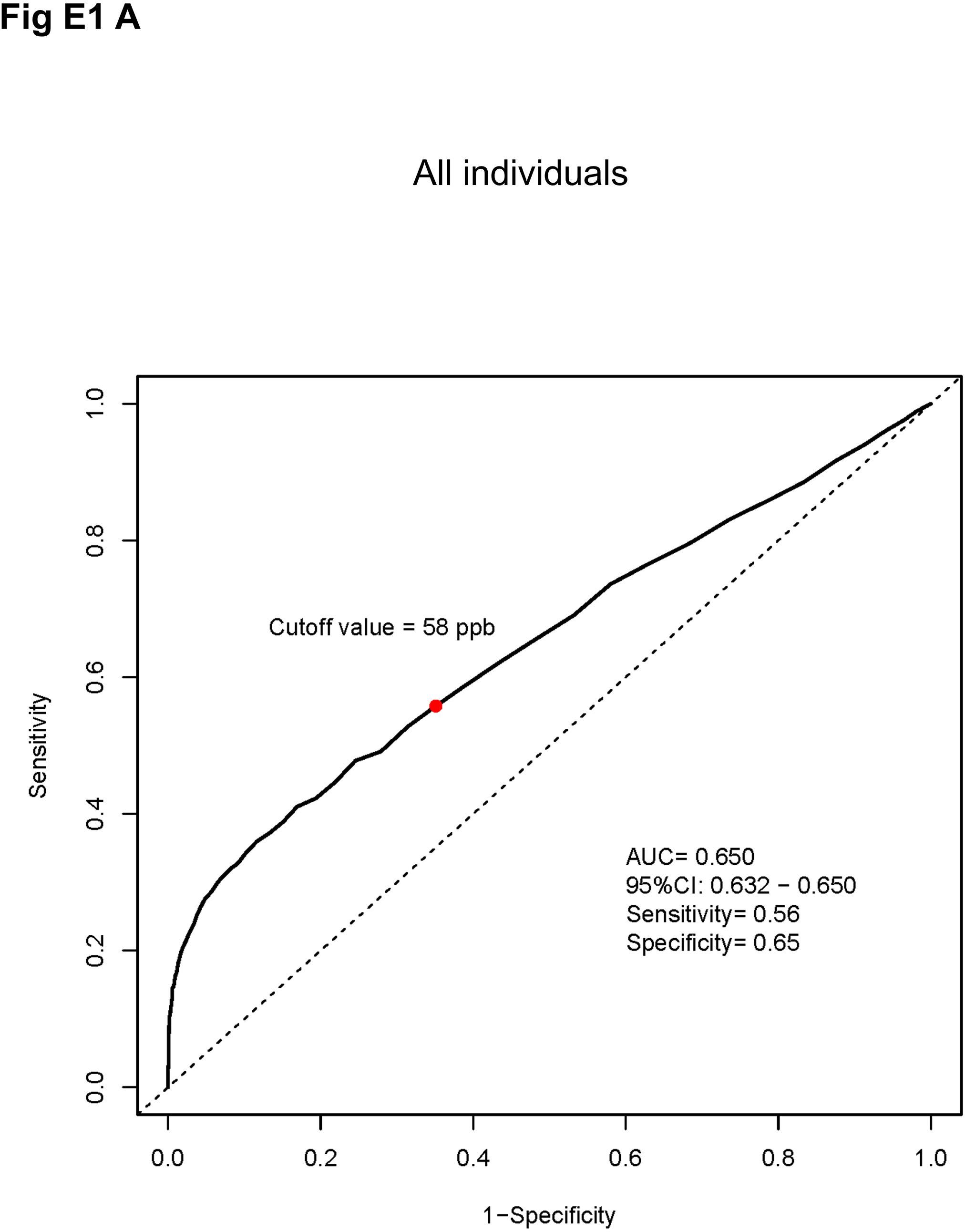

Supplement: Supplementary Fig E1 A [file figs1.jpg]

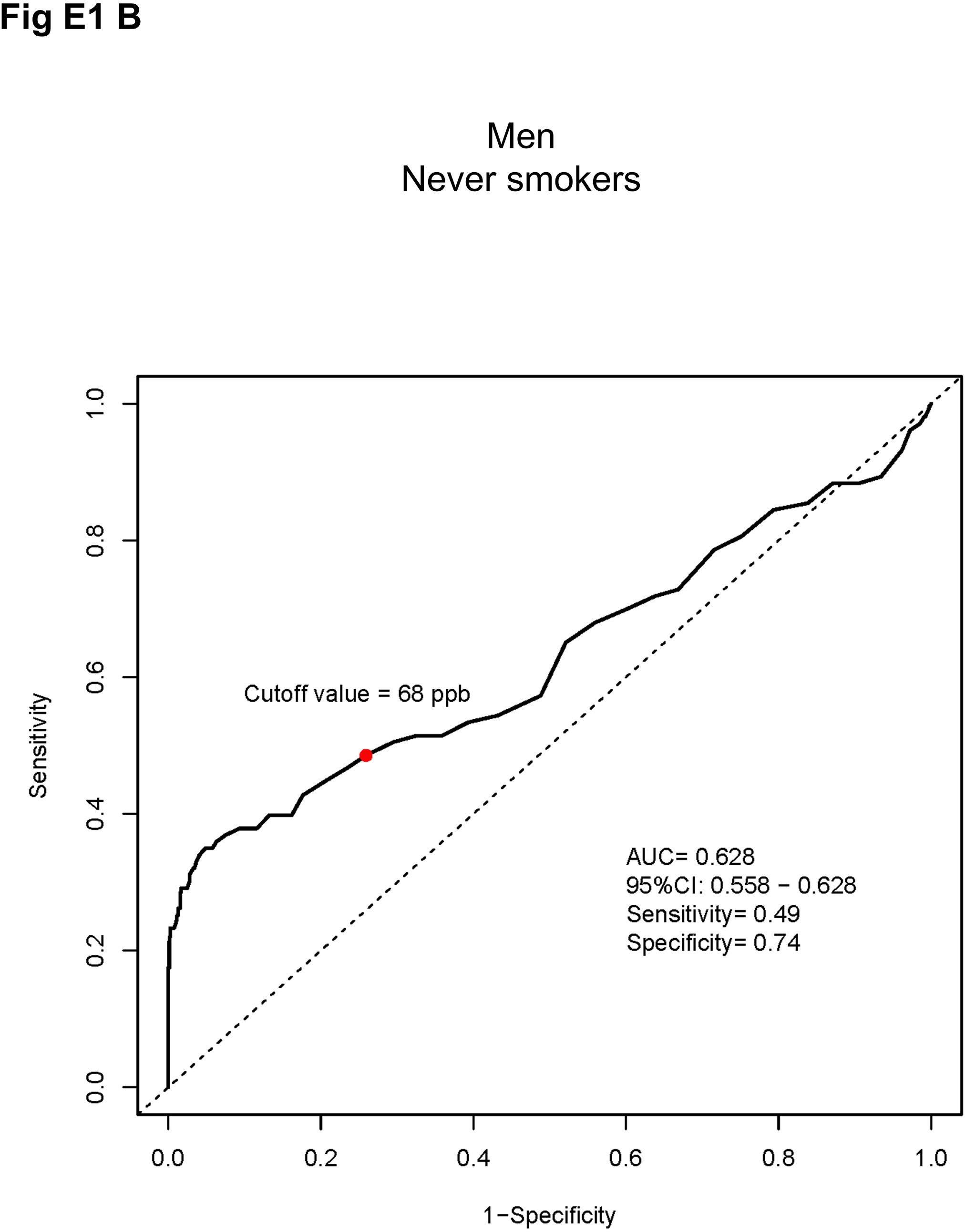

Supplement: Supplementary Fig E1 B [file figs2.jpg]

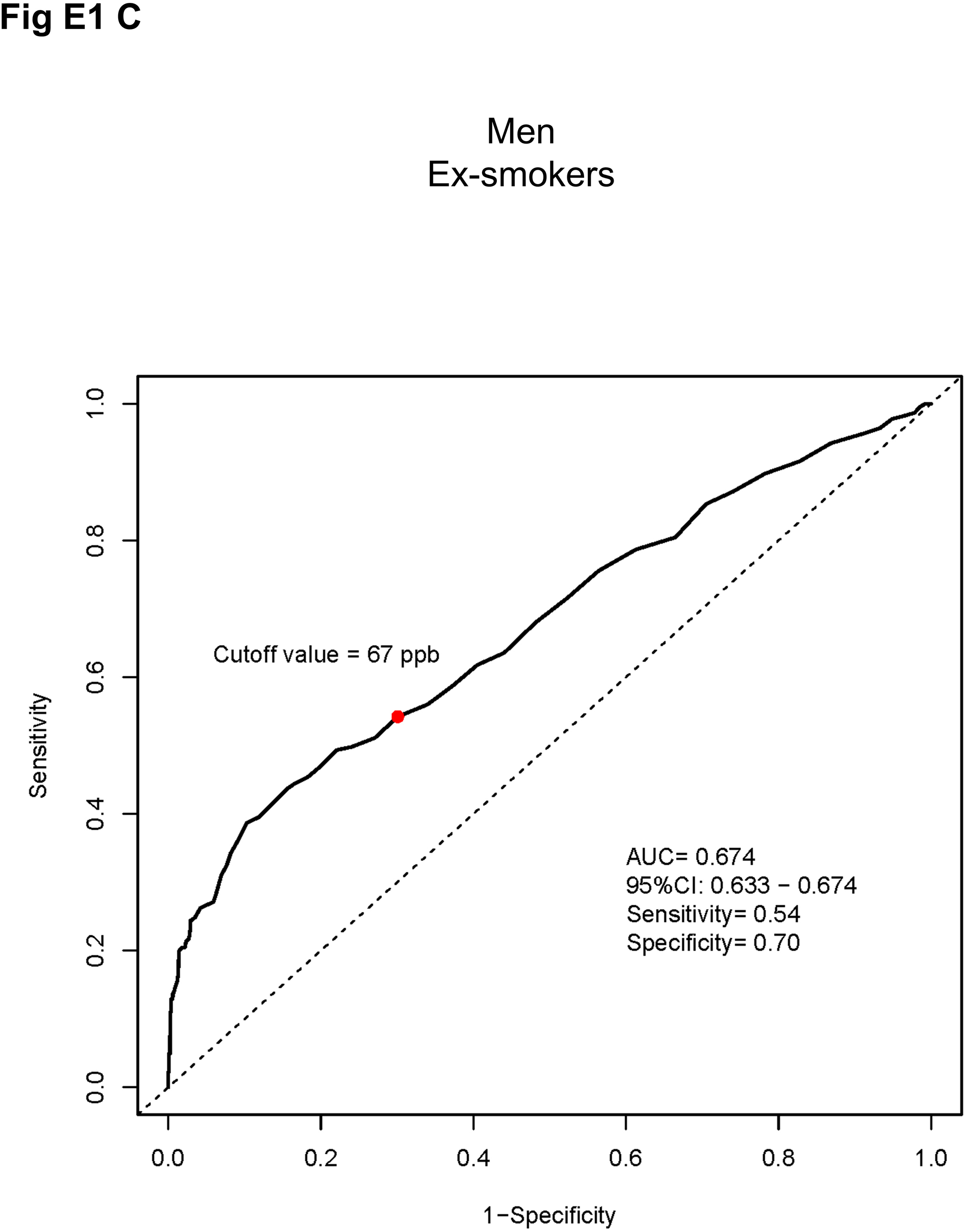

Supplement: Supplementary Fig E1 C [file figs3.jpg]

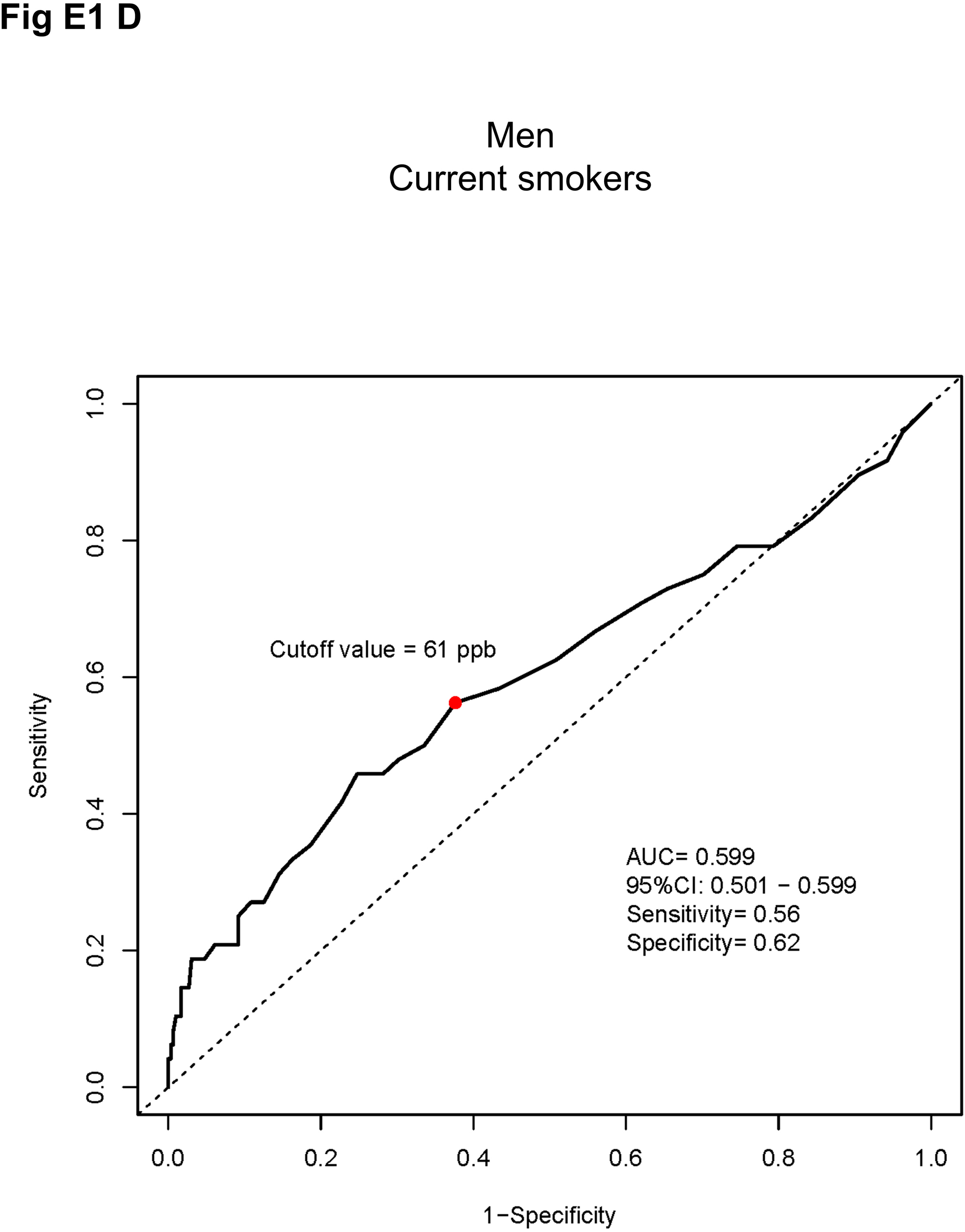

Supplement: Supplementary Fig E1 D [file figs4.jpg]

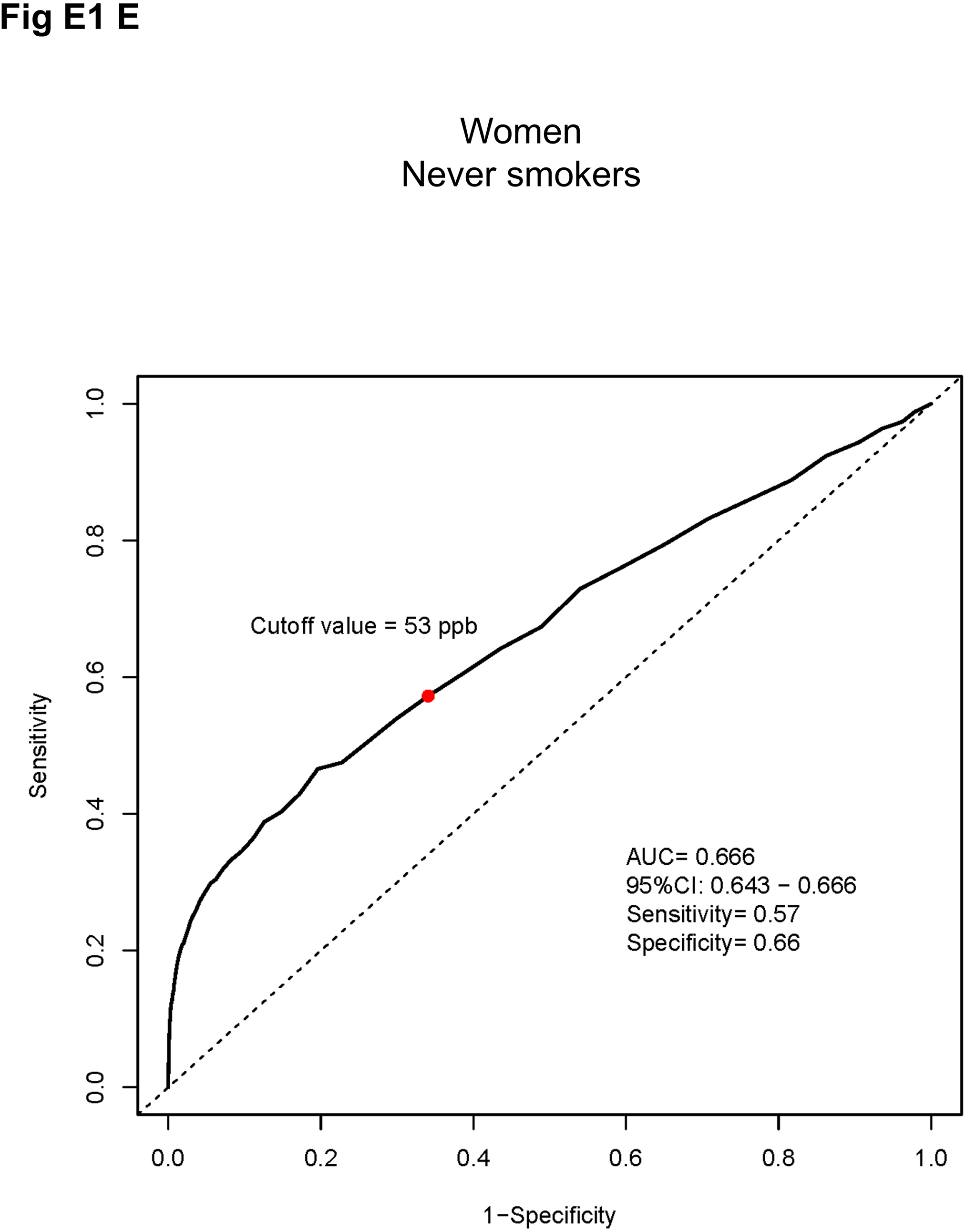

Supplement: Supplementary Fig E1 E [file figs5.jpg]

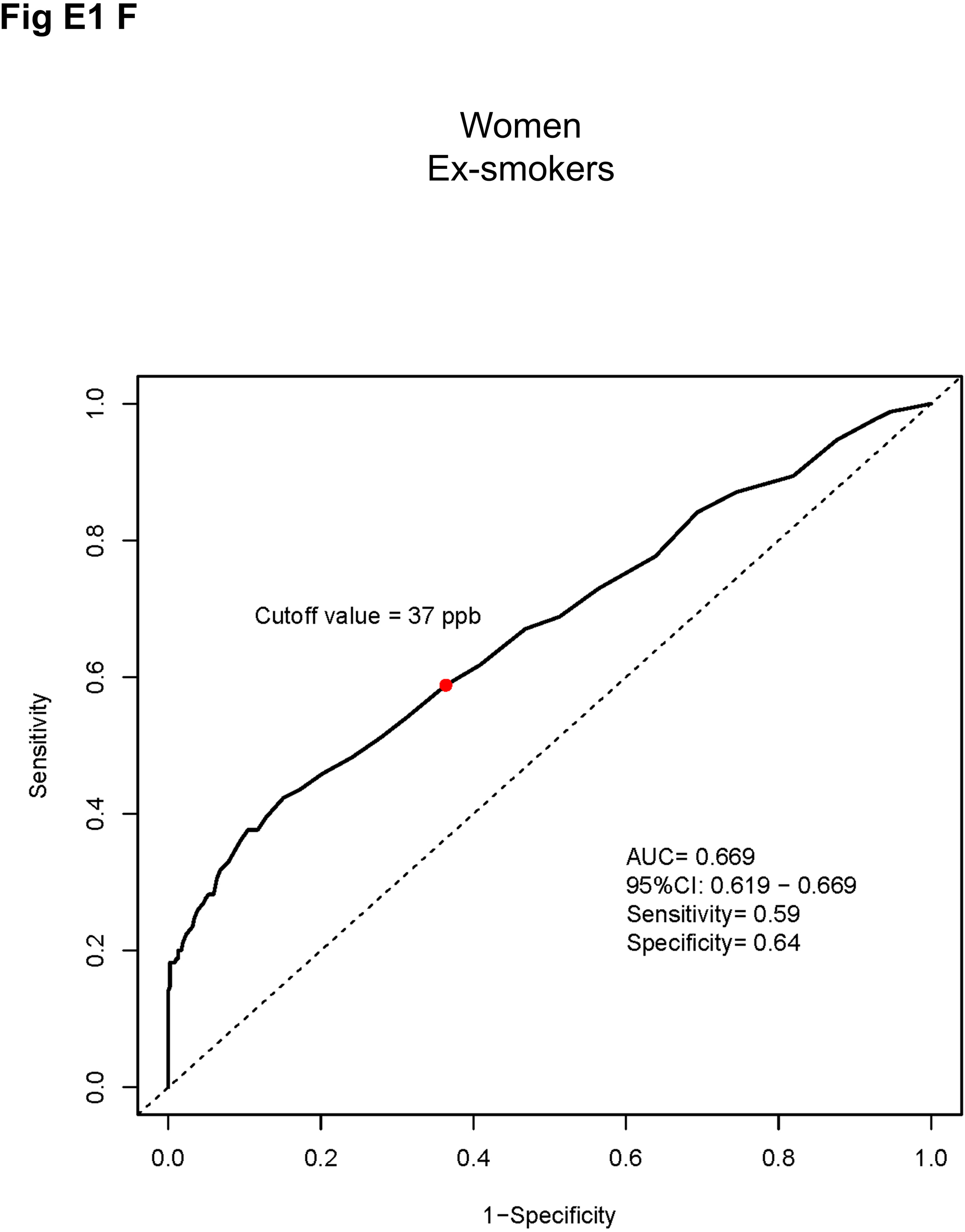

Supplement: Supplementary Fig E1 F [file figs6.jpg]

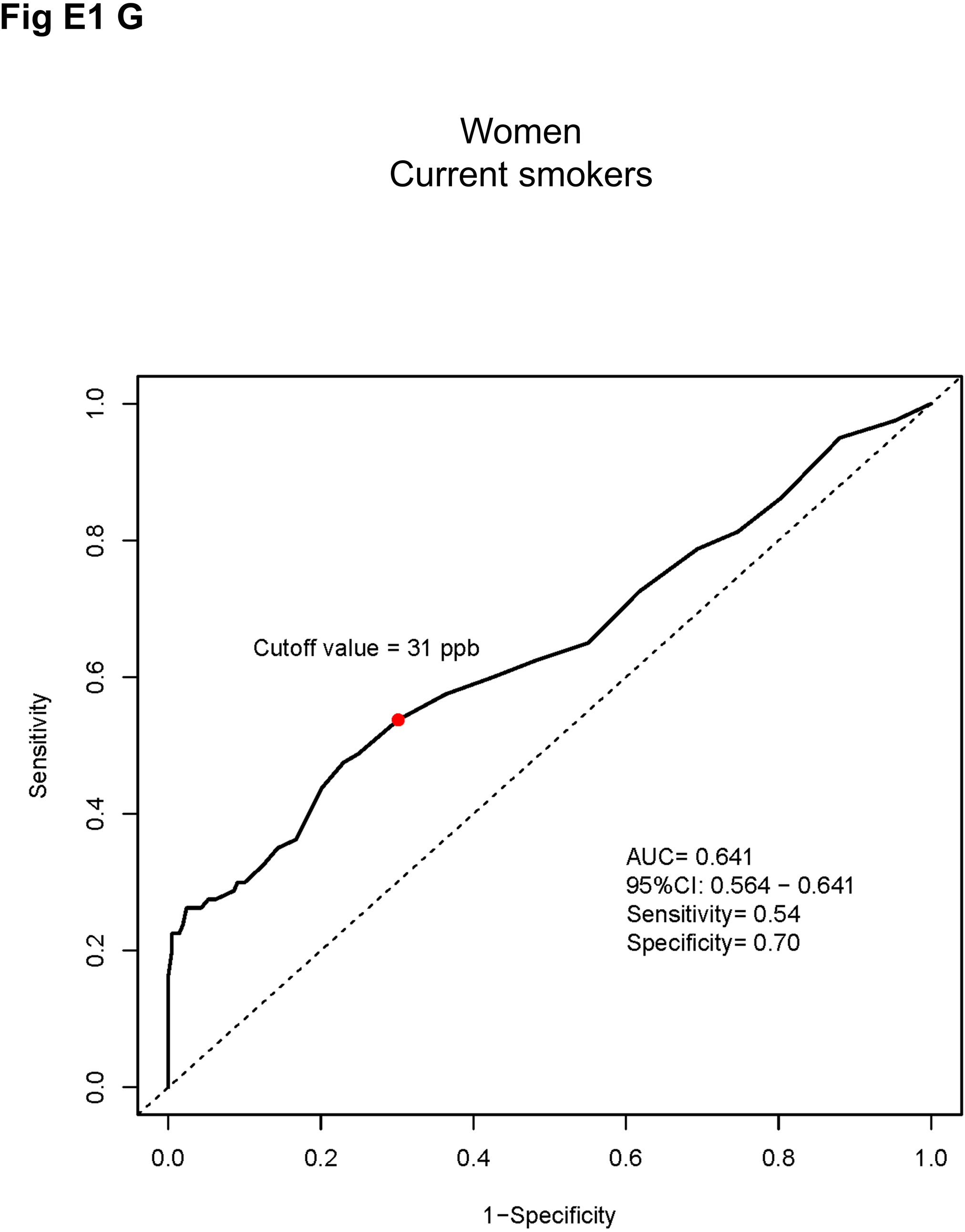

Supplement: Supplementary Fig E1 G [file figs7.jpg]
