# Supplementary material for: Fractional exhaled nitric oxide distribution and its relevant factors in the general adult population and its healthy subpopulation
Source: J Allergy Clin Immunol Glob. 2024 Apr 8;3(3):100253. doi: 10.1016/j.jacig.2024.100253 (PMC11090912; doi:10.1016/j.jacig.2024.100253)
Supplement: Supplementary data [file mmc1.docx]

**Supplemental material**

**FeNO distribution and its relevant factors in the general adult population and its 'healthy' subpopulation**

**DETAILED METHODS**

**Study design and subjects**

This cross-sectional study was conducted using data from the TMM CommCohort Study; details of the TMM CommCohort Study have been published in previous papers^1,2^. Briefly, this cohort study aimed to contribute to the development of personalized healthcare and medicine worldwide, for which many genomic and epidemiological studies have been conducted including a genome-wide association study for FeNO ^3^. Three approaches were used to recruit participants: a type 1 survey (40,433 participants) was conducted at specific community health screening sites; an additional type 1 survey (664 participants) was conducted on different dates at specific community health screening sites; and a type 2 survey (13,855 participants) was conducted at a community support center. The study included men and women aged 20 years and older living in Miyagi Prefecture, northeastern Japan. The survey and recruitment took place between May 2013 and March 2016. Informed consent was obtained from 54,952 participants. After the baseline survey, a repeat assessment of the center-based survey was conducted during the second period from June 2017 to March 2021. Those who participated in the baseline survey were invited by mail to participate in the repeat assessment survey. The study was approved by the Institutional Ethics Committee of the Tohoku Medical Megabank Organization (initial approval date: 10 May 2013 (approval number: 2012-4-617); latest update date: 30 August 2023 (approval number: 2023-4-076)).

This study only used data from 29,383 people who underwent FeNO measurement and spirometry at a repeat assessment. Those who withdrew from the study by 17 January 2022, those who did not return the self-report questionnaire, those who did not undergo lung function measurements, and those with missing data on height, weight, smoking history, white blood cell count, peripheral blood eosinophil count, specific IgE to house dust mite (*Dermatophagoides farinae* (*D. farinae*)), and specific IgE to cedar pollen were excluded (n=3,457). Therefore, only data from 25,926 participants were analysed.

**Assessment of FeNO**

FeNO was measured using a NIOX VERO® (Circassia AB, Sweden). Measurements were performed according to the measurement guidelines jointly proposed by the American Thoracic Society (ATS) and the European Respiratory Society (ERS) ^4^ and the official statement of the Japanese Respiratory Society (JRS) for FeNO measurement and interpretation^5^. All subjects abstained from food and drink for one hour prior to FeNO measurement. Measurements were taken with continuous expiration for 10 seconds at an expiratory flow rate of 50 mL/s.

We examined the distribution of FeNO in (1) all participants, (2) the healthy subpopulation 1; the participants without a history of airway diseases (asthma, COPD, chronic bronchitis, other respiratory diseases, allergic rhinitis and chronic sinusitis), (3) the healthy subpopulation 2; the participants without the history of airway diseases, a restrictive or obstructive ventilatory defect, and positivity for type 2 biomarkers (specific IgE to house dust mite and cedar pollen and elevated blood eosinophil count (≥150 µL^-1^)).

FeNO levels were categorized into the following four groups according to ATS/ERS ^6^ and JRS^5^ guidelines; <25 ppb, 25-34 ppb, 35-49 ppb and ≥50 ppb. Subjects with FeNO ≥35 ppb were defined as the elevated FeNO group using the cut-off considered appropriate for the ancillary diagnosis of asthma according to previous articles ^5,7^.

**Assessment of lung function**

Lung function parameters, including FEV_1_, FVC and VC, were measured using a spirometer (HI-801; Chest M.I., Incorporation, Japan). Spirometry was performed in the sitting position with a nose clip attached. To calculate the percentage of predicted FEV_1_ or VC (%FEV_1_ or %VC), which are values of lung function measurements adjusted for age, gender, and height, we used the reference spirometry values for Japanese adults calculated using the LMS (Lambda Mu and Sigma) method ^8^. A restrictive ventilatory defect was defined as %VC less than 80%. An obstructive ventilatory defect was defined as an FEV_1_/FVC ratio less than 0.7.

**Other measurements**

A self-administered questionnaire was used to assess demographic characteristics, smoking status and current medical history, including asthma, COPD, chronic bronchitis, other respiratory diseases, allergic rhinitis, chronic sinusitis and atopic dermatitis. Age was determined at the time of the visit to the community support center. Smoking status was classified into three categories: never smoker, ex-smoker and current smoker. Never smokers were defined as those who had smoked fewer than 100 cigarettes in their lifetime. Ex-smokers were defined as those who had smoked more than 100 cigarettes in their lifetime but were not currently smoking. Current smokers were defined as participants who had smoked at least 100 cigarettes in their lifetime and reported being current smokers.

Height was measured to the nearest 0.1 cm using a stadiometer (AD6400; A&D Co., Ltd., Tokyo, Japan). Weight was measured in 0.1 kg increments, and 1.0 kg was subtracted to account for the weight of the participant's clothing using a body composition analyzer (InBody720; Biospace Co., Ltd., Seoul, Korea). BMI was calculated as weight (kg) divided by height [meters squared (m^2^)].

Complete blood count and differential count were measured using a certified automated analyzer. Cedar pollen-specific IgE and house dust mite (*D. farinae*)-specific IgE were detected by fluorescence enzyme immunoassay. Cedar pollen-specific IgE was measured because cedar pollen is the most common allergen of seasonal allergic rhinitis in Japan. The reason for measuring *D. farinae* -specific IgE was that it was the allergen with the highest positive rate in a previous study of Japanese asthmatic patients ^9^.

**Statistical analysis**

Data are presented as mean (standard deviation [SD]) or median (interquartile range [IQR]) for continuous variables and as number (%) for categorical variables. Data are presented for all participants and for subgroups categorized by current asthma history and smoking status. For the characteristics of the four subgroups categorized by FeNO, a trend test was performed for continuous variables using a simple linear model to assess linear association. We also performed a chi-squared test to compare the characteristics of categorical variables between the FeNO subgroups.

To analyze the association between elevated FENO (FeNO >35 ppb) and various variables, multivariate logistic analysis was performed to calculate odds ratios (ORs) and 95% confidence intervals (CIs). The following covariates were included in the study: age (<30, 30-39, 40-49, 50-59, 60-69, 70-79, ≥80), gender, height, weight (or underweight [BMI < 18.5] or overweight [BMI ≥25.0), smoking status, restrictive ventilatory defect, obstructive ventilatory defect, peripheral blood eosinophil count (<150 of eosinophils, 150-299 of eosinophils, ≥300 of eosinophils), specific IgE positivity to house dust mite, specific IgE positivity to cedar pollen, current history of asthma, COPD, other respiratory diseases, allergic rhinitis, chronic sinusitis, atopic dermatitis, hypertension, diabetes mellitus, dyslipidemia, hyperuricemia, stroke, and myocardial infarction.

Receiver Operating Characteristics (ROC) analysis was performed to investigate the cut-off value of FeNO that discriminates self-reported asthmatics from healthy subjects (healthy subpopulation 2). The cutoff value is determined by the point on the ROC curve which is closest to the top left.

*P* <0.05 was considered significant. All analyses were performed with R software version 4.1.2 (R Foundation for Statistical Computing, Vienna, Austria).

**REFERENCES**

1. Hozawa A, Tanno K, Nakaya N, et al. Study Profile of the Tohoku Medical Megabank Community-Based Cohort Study. *J. Epidemiol.* 2021;31(1):65-76.

2. Kuriyama S, Yaegashi N, Nagami F, et al. The Tohoku Medical Megabank Project: Design and Mission. *J. Epidemiol.* 2016;26(9):493-511.

3. Yamada M, Motoike IN, Kojima K, et al. Genetic loci for lung function in Japanese adults with adjustment for exhaled nitric oxide levels as airway inflammation indicator. *Communications Biology.* 2021;4(1):1288.

4. ATS/ERS recommendations for standardized procedures for the online and offline measurement of exhaled lower respiratory nitric oxide and nasal nitric oxide. *Am. J. Respir. Crit. Care Med.* 2005;171(8):912-930.

5. Matsunaga K, Kuwahira I, Hanaoka M, et al. An official JRS statement: The principles of fractional exhaled nitric oxide (FeNO) measurement and interpretation of the results in clinical practice. *Respir Investig.* 2021;59(1):34-52.

6. Dweik RA, Boggs PB, Erzurum SC, et al. An official ATS clinical practice guideline: interpretation of exhaled nitric oxide levels (FENO) for clinical applications. *Am. J. Respir. Crit. Care Med.* 2011;184(5):602-615.

7. Matsunaga K, Hirano T, Akamatsu K, et al. Exhaled nitric oxide cutoff values for asthma diagnosis according to rhinitis and smoking status in Japanese subjects. *Allergol Int.* 2011;60(3):331-337.

8. Kubota M, Kobayashi H, Quanjer PH, Omori H, Tatsumi K, Kanazawa M. Reference values for spirometry, including vital capacity, in Japanese adults calculated with the LMS method and compared with previous values. *Respiratory Investigation.* 2014;52:242-250.

9. Fukutomi Y, Kawakami Y, Taniguchi M, et al. Allergenicity and Cross-Reactivity of Booklice (Liposcelis bostrichophila): A Common Household Insect Pest in Japan. *Int. Arch. Allergy Immunol.* 2012;157(4):339-348.

**Supplemental Table**

**Table S1** The cutoff values of FeNO that distinguish asthma from healthy subjects by receiver operating characteristics (ROC) analyses using data from both the 8321 individuals of healthy subpopulation 2 and the 1386 individuals who self-reported asthma.

|  | Cutoff value (ppb) | AUC (95% CI) | Sensitivity | Specificity |
| --- | --- | --- | --- | --- |
| All | 58 | 0.650 (0.632-0.650） | 0.56 | 0.65 |
| Men |  |  |  |  |
| Never-smoker | 68 | 0.628 (0.558-0.628) | 0.49 | 0.74 |
| Ex-smoker | 67 | 0.674 (0.633-0.674) | 0.54 | 0.7 |
| Current smoker | 61 | 0.599 (0.501-0.599) | 0.56 | 0.62 |
| Women |  |  |  |  |
| Never-smoker | 53 | 0.666 (0.643-0.666) | 0.57 | 0.66 |
| Ex-smoker | 37 | 0.669 (0.619-0.669) | 0.59 | 0.64 |
| Current smoker | 31 | 0.641 (0.564-0.641) | 0.54 | 0.7 |

AUC, area under curve; CI, confidence interval.

**Supplemental Figure Legend:**

**Supplemental Figure Fig S1.** Receiver operating characteristics (ROC) analyses using data from both the 8321 individuals of healthy subpopulation 2 and the 1386 individuals who self-reported asthma. The cutoff values of FeNO that distinguish asthmatics from healthy subjects were shown with area under curve (AUC), 95% confidence interval (CI) of AUC, sensitivity, and specificity. ROC analyses were performed in all participants (A), men never smokers (B), men ex-smokers (C), men current smokers (D), women never smokers (E), women ex-smokers (F), women current smokers (G).

**
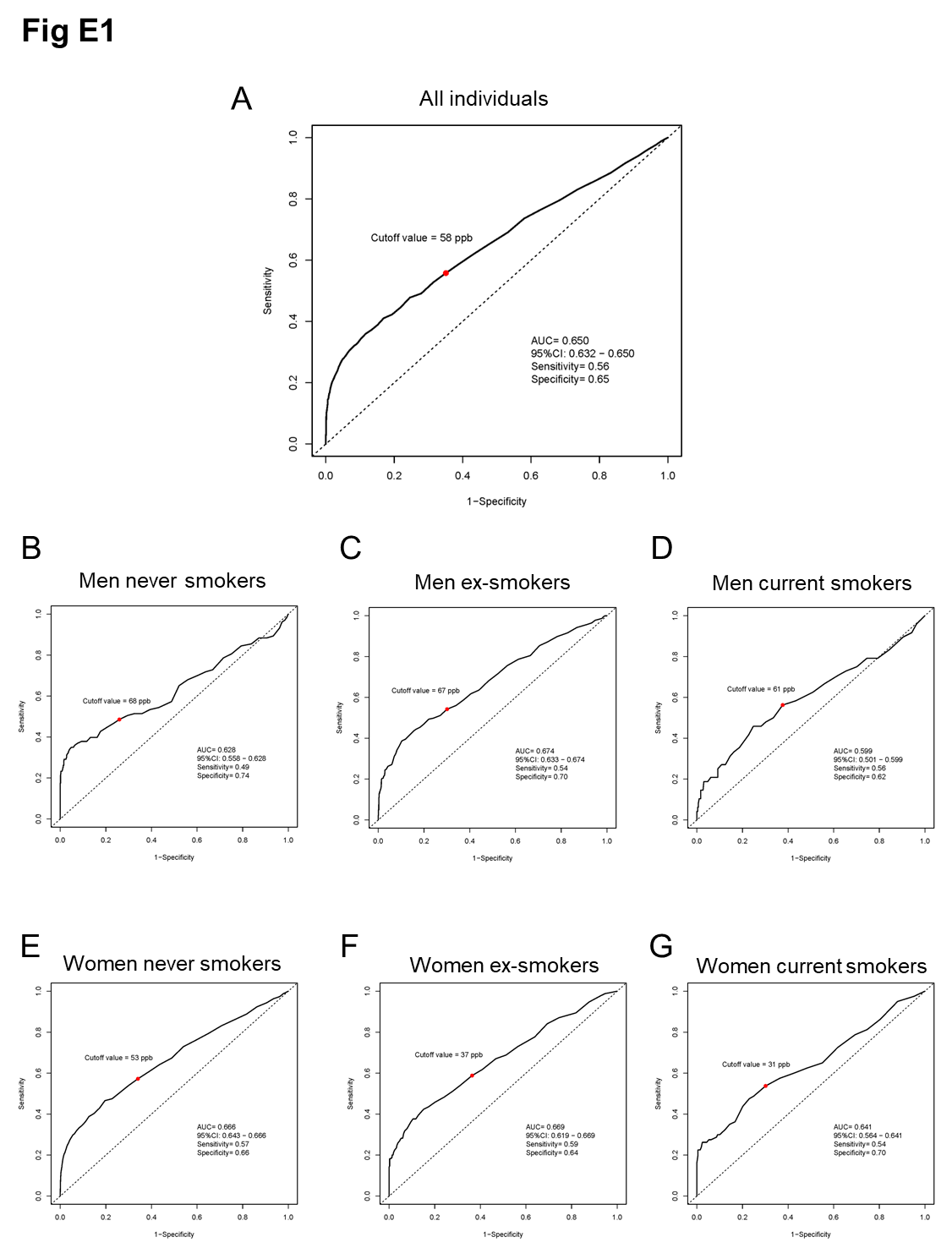
**
